# Is estrogen receptor negative breast cancer risk associated with a fast life history strategy?

**DOI:** 10.1093/emph/eov034

**Published:** 2016-01-18

**Authors:** Brandon H. Hidaka, Amy M. Boddy

**Affiliations:** ^1^Department of Dietetics and Nutrition, University of Kansas Medical Center, Kansas City, KS 66205, USA,; ^2^Department of Psychology, Arizona State University, Tempe, AZ 85287, USA and; ^3^Center for Evolution and Cancer, University of California San Francisco, San Francisco, CA USA

Risk factors for breast cancer are often confusing and contradictory. Discrepancies are likely due to different subtypes having divergent risk factors. An important distinction between breast cancer subtypes is hormone-receptor status. Compared to women diagnosed with estrogen receptor positive (ER+) breast cancer, those with estrogen receptor negative (ER−) tumors are usually diagnosed at a younger age and have a higher mortality [1]. Few studies have attempted to explain ‘why’ breast cancer subtypes have different risk factors.

In a recent meta-analysis, Aktipis *et al.* [2] demonstrated that modern reproductive behaviors are more strongly associated with the development of ER+ than ER− breast cancer. The systematic review specifically reported the following: (a) fewer total offspring is a risk factor for ER+, but not ER−, tumors; (b) older age at first birth is associated with risk for ER+, but not ER−, tumors and (c) lower age of menarche is a risk factor for both ER+ and ER− tumors. These results support the evolutionary mismatch hypothesis for ER+ breast cancer susceptibility; that is, modern women have a higher risk from more menstrual cycles and greater cumulative exposure to estrogen compared to ancestral humans. However, modern reproductive patterns seem to have little influence on hormone-independent breast cancer risk. Breast cancer susceptibility may require complementary evolutionary explanations. In this article, we propose that the risk factors for ER− breast cancer, low socioeconomic status (SES), poor diet and early age of menarche are features of a faster life history strategy.

**Figure 1.**
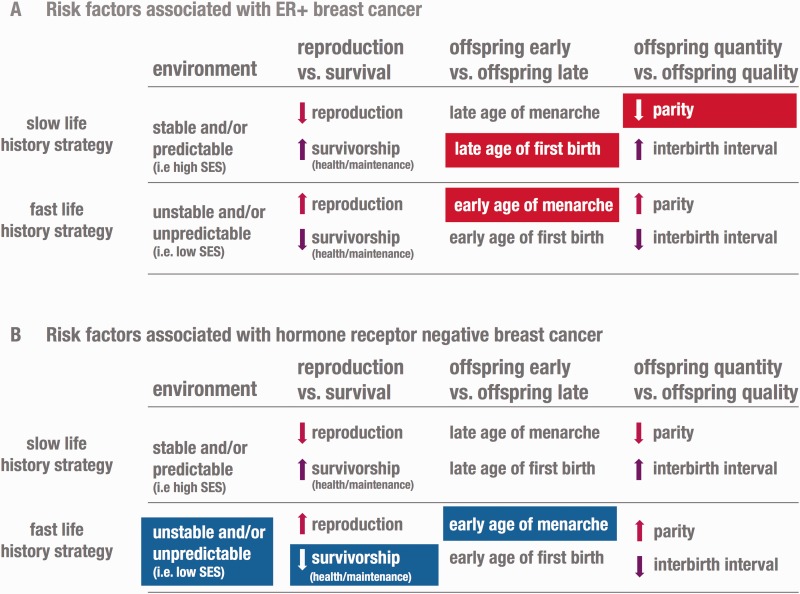
Risk factors associated with ER+ breast cancer include low parity, late age of first birth and early age of menarche. These reproductive traits all increase a woman’s exposure to estrogen and support the modern mismatch hypothesis. In contrast, risk factors for hormone negative breast cancer include early age of menarche, low SES and decreased consumption of foods rich in micronutrients (i.e. less behavioral investment in somatic maintenance).

Life history theory provides a framework for understanding how, when and why organisms allocate their resources [3]. To maximize reproductive success, organisms must strategically distribute resources toward growth, reproduction and somatic maintenance. This process of phenotypic development is largely determined by trade-offs. Three of the most important trade-offs include reproduction versus survival (i.e. growth and somatic maintenance), offspring now versus offspring later and offspring quality versus offspring quantity (Fig. 1).

Environmental cues during critical periods of development help to guide an organism’s life history strategy. Environments with high extrinsic mortality and unpredictable resources tend to select individuals with a fast life history strategy, that is, mature and invest in reproduction earlier at the cost of growth and/or somatic maintenance [4]. In contrast, secure, predictable environments delay reproduction and promote investment in growth and maintenance, characteristics of a slow life history strategy. Tumor suppression is a major component of somatic maintenance; this includes DNA repair, cycle control and immune function [3]. Individuals that distribute resources to reproduction at the cost of somatic maintenance may increase their risk for cancer through less investment in DNA repair (leading to higher mutation rates) and/or immunosurveillance.

Accordingly, individuals with characteristics of a fast life history strategy may have a fertility advantage early in life that is accompanied by increased cancer risk later in life. There is preliminary evidence that suggests women at elevated risk for hormone-independent breast cancer have a different reproductive profile. Women who carry a germline mutation in *BRCA1* or *BRCA2* are more likely than non-carriers to develop an ER− breast tumor [5]; carriers of these mutations were recently reported to have significantly more children, shorter birth intervals and end their child-bearing years later than aged-matched controls [6]. Although further research is needed to confirm this observation, it is one of the first report to demonstrate a genetic link between trade-offs in reproduction and breast cancer.

If women at risk for hormone-independent breast cancer invest more in reproduction, we should expect to see evidence for less investment in somatic maintenance. An individual’s overall cancer risk, including breast cancer, increases over their lifetime. One of the most important epidemiologic patterns of breast tumor subtypes is that ER− tumors are more common among younger women [1]. One potential explanation for the earlier age of onset of ER− breast cancer could be faster biological aging due to less somatic maintenance.

Environmental cues, such as resource predictability and extrinsic mortality, impact an individual’s life strategy. SES provides an indicator of an individual’s environment and resources. Interestingly, incidence of breast cancer subtypes varies along the SES gradient. Compared to US women with other breast cancer subtypes, those diagnosed with a hormone-independent tumor are more likely to report Black or Hispanic ethnicity (versus non-Hispanic White) [1]. And, statistically independent of race and ethnicity, ER− tumors are more common among women of lower SES [1]. These patterns suggest that women from less privileged backgrounds have a higher relative risk of ER− breast cancer compared with women that are (on average) reared in more stable and predictable environments.

As mentioned above, early environmental cues can steer an organism’s life history strategy. In humans, age at first menstruation signals the switch from investment in growth and/or maintenance to reproduction. In modern-industrialized samples, age of menarche can be delayed by family warmth and paternal investment, while pubertal timing can be accelerated in moderately stressful environments of nutritional adequacy [7]. In USA, African American girls begin puberty earlier than White counterparts [7]. An early age of menarche is associated with a higher risk of ER+ and ER− breast cancer [2]. A low SES environment in childhood may be stressful enough to prompt an accelerated life history strategy.

Eating nutritious food and leisure-time physical activity are examples of behavioral somatic maintenance. Individuals of low SES report investing less in their health; interestingly, this association was found to be completely mediated by perceived extrinsic mortality risk [8]. Therefore, beyond economic barriers, high rates of violence in low SES neighborhoods may contribute to decreased engagement health-promoting behavior. A pooled analysis of nearly 1 million women followed for 11–20 years found that those who ate more fruits and vegetables were less likely to develop ER− tumors compared with those who ate fewer fruits and vegetables [9]. This effect was statistically independent of ethnicity, education and 14 other potentially confounding risk factors (e.g. alcohol consumption, body mass index and hormone replacement therapy) [9]. Surprisingly, fruit and vegetable consumption was not found to be protective against ER+ breast tumors. A similar pattern is observed for carbohydrate consumption; postmenopausal women with a higher dietary glycemic load have an elevated risk of ER− breast cancer, but not ER+ breast cancer [10]. Poor diet quality is more strongly associated with ER− than with ER+ breast cancer.

Threatening stimuli early in life can increase the risk of adult-onset diseases via epigenetic changes [11]. Although much of this literature is not framed evolutionarily, these epigenetic profiles theoretically guide resources away from somatic maintenance, that is, a faster life history strategy. Somatic maintenance is costly and genes involved in tumor suppression are frequently downregulated via epigenetic silencing in breast tumors [12]. Stress-induced epigenetic changes may in turn be exacerbated by a low intake of fruits and vegetables, which are rich sources of micronutrients and other bioactive compounds. Food components can influence epigenetic processes multitudinously; for example, several vitamins participate in the methyl cycle, thereby affecting DNA methylation, and several phytochemicals are known to directly modulate histone acetylation processes [13, 14]. We suggest that epigenetic alterations may be a mechanism that links diet, the SES gradient and ER− breast cancer risk [15].

The proposed connection between life history strategy and ER− breast cancer risk implies a few immediate predictions. Early life stressors that accelerate pubertal timing may be associated with increased promoter methylation of tumor suppressor genes, including those expressed in human breast epithelium. Women with hormone-independent breast cancer often have *BRCA1* promoter hypermethylation [12]. Information on early environment (SES), reproductive traits (pubertal timing, age of first birth and number of offspring) and epigenetic profiles could provide insight into the relationship between life history strategy and breast cancer susceptibility. We also predict that other activities related to low behavioral somatic maintenance, for example, alcohol consumption, smoking and body fatness, would be associated with elevated hormone-independent breast cancer risk. Although it will be difficult to untangle the effects of alcohol and body fatness on breast cancer risk because both directly increase estrogen levels [16]. It is also unclear how life history trade-offs in the host affect trade-offs in the tumor. *In vitro* studies could help determine how ER+ and ER− breast cancer cells differ in life history characteristics, such as division rate and death rate. Another challenge in this domain of research is the heterogeneous expression of estrogen-receptor both within and between tumors categorized as ER+. How this widespread methodology affects our observations cannot currently be measured; the question would best be interrogated by comparing risk factor differences between breast tumor subtypes from various categorization schemes. Lastly, if hormone-independent breast cancer is a result of less somatic maintenance, we would predict these tumors to have greater genetic heterogeneity (via a higher mutation rate) and the individual to have a decreased immune function, which is consistent with ER− tumors’ more aggressive phenotype.

In summary, there appear to be different risk factors for different types of breast cancer. Greater cumulative estrogen exposure from delayed childbirth and fewer total offspring is associated with ER+ breast cancer, a cost of modern reproductive behavior. However, hormone-independent breast tumors exhibit a different profile of environmental risk factors. ER− breast cancer susceptibility shows no association with parity or age at first birth. Instead, genetic variants associated with higher fertility, racial and ethnic minority membership, low SES and eating fewer fruits and vegetables (behavioral somatic maintenance) are associated with a higher risk for ER− breast cancer. An accelerated life history strategy lends a framework for explaining these associations that will hopefully lead to clinical application, but at this stage, more mechanistic evidence is needed. Future research into the epigenetic effects of early psychosocial stress and malnutrition could enhance understanding of why some women are more vulnerable to the most lethal subtype of breast cancer.
